# Screening of Salivary Biomarkers of Bisphosphonate-Related Osteonecrosis of the Jaw in a Diabetic Rat Model

**DOI:** 10.3390/cimb47121002

**Published:** 2025-11-28

**Authors:** Ke Qin, Masato Nakagawa, Yoichi Sumi, Baiyan Zhang, Mamoru Uemura, Yoshitomo Honda

**Affiliations:** 1 Department of Oral Anatomy, Osaka Dental University, 8-1 Kuzuhahanazono-cho, Hirakata 573-1121, Osaka, Japan; tn_will@outlook.com (K.Q.); honda-y@cc.osaka-dent.ac.jp (Y.H.); 2 Department of Anatomy, Osaka Dental University, 8-1 Kuzuhahanazono-cho, Hirakata 573-1121, Osaka, Japan; sumi-y@cc.osaka-dent.ac.jp (Y.S.); mamoru-u@cc.osaka-dent.ac.jp (M.U.); 3 Department of Geriatric Dentistry, Osaka Dental University, 8-1 Kuzuhahanazono-cho, Hirakata 573-1121, Osaka, Japan; baeky0613@163.com

**Keywords:** saliva, biomarkers, bisphosphonate-related osteonecrosis of the jaw (BRONJ), diabetes, salivary gland, tooth extraction, streptozotocin

## Abstract

Diabetes is a significant risk factor for bisphosphonate-related osteonecrosis of the jaw (BRONJ), a severe oral complication with limited treatment options. Salivary testing offers a noninvasive approach for monitoring BRONJ risk; however, few studies have investigated salivary biomarkers in BRONJ. This study screened salivary biomarkers that reflect the progression of BRONJ under diabetic conditions. A diabetic BRONJ rat model was established to screen for diabetes-related biochemical biomarkers in saliva. Streptozotocin (STZ) administration elevated blood glucose and glycated albumin levels and altered lipid and renal function markers, confirming diabetes induction. Subsequent zoledronic acid (ZA) administration and extraction of the maxillary first molar delayed epithelialization, inflammatory cell infiltration, bone exposure, and necrosis in extraction sockets, indicating successful establishment of a diabetic BRONJ model. This model showed reductions in submandibular and sublingual gland size, as well as in acinar cell number. Although salivary secretion volume was reduced, saliva samples were successfully collected from all groups. Screening identified elevated urea nitrogen (UN) and total ketone bodies (T-KB) in the STZ + ZA group. These findings suggest that salivary UN and T-KB may reflect disease progression and serve as potential biomarkers for predicting BRONJ risk under diabetic conditions.

## 1. Introduction

Diabetes is a chronic metabolic disease that causes various systemic complications and has become a major global health issue [1,2]. Persistent hyperglycemia induces chronic inflammation and oxidative stress, which are further exacerbated by abnormalities in lipid metabolism and renal dysfunction [3,4,5]. Consequently, diabetes leads to serious complications, including the three major complications—retinopathy, nephropathy, and neuropathy—as well as atherosclerosis, cardiovascular disease, and reduced resistance to infection.

Diabetes is also recognized as a major risk factor for bisphosphonate-related osteonecrosis of the jaw (BRONJ), a serious condition characterized by bone exposure and necrosis [6]. Although results vary among studies, numerous clinical investigations have reported diabetes as a risk factor for BRONJ development [7,8]. However, because BRONJ is a multifactorial disorder with a low incidence, human studies face significant limitations. Consequently, research has also been conducted using diabetic rodent models of BRONJ [9,10]. Most previous studies have focused on drug metabolism, immunological and dermatological mechanisms, or inflammatory pathways, whereas only a limited number have investigated saliva or the salivary glands (SGs) in the context of BRONJ [11,12,13].

The identification of diagnostic markers to assess the risk of BRONJ remains a crucial challenge. Several studies have analyzed blood-based biomarkers to monitor disease progression and metabolic status [14,15]. Although several bone markers have been considered promising, no clear consensus has been established [16]. Moreover, glycemic control status is considered a critical factor in assessing the risk of oral complications, including localized infections and BRONJ [17].

In recent years, saliva has garnered attention as a biological sample comparable to blood and urine because of its noninvasive and easy-to-collect properties [18,19]. Saliva contains diverse components, including metabolic markers that reflect glucose and lipid metabolism, inflammatory cytokines, and molecules associated with oxidative stress [20,21,22]. The usefulness of salivary biomarkers in diabetes has been previously reported [23,24]. Moreover, because saliva directly reflects the local oral environment, it serves as a valuable diagnostic fluid for assessing the pathological status of jawbone and periodontal tissues [21,25]. However, to the best of our knowledge, no studies have investigated diabetes-related biochemical factors in saliva in a BRONJ model with diabetes. This study aimed to determine whether systemic metabolic alterations caused by diabetes under BRONJ conditions are reflected in salivary components, thereby identifying potential salivary biomarkers associated with disease progression.

## 2. Materials and Methods

### 2.1. Animal Experiment

Four-week-old male Wistar rats were purchased from Shimizu Experimental Materials Co., Ltd. (Kyoto, Japan). All animals were housed in a well-maintained animal facility under standard experimental conditions throughout the study period. All experimental procedures were approved by the Osaka Dental University Institutional Animal Care and Use Committee (Approval No.: 25-03007).

### 2.2. Procedure for Establishing a Diabetic BRONJ Model

**Figure 1:**
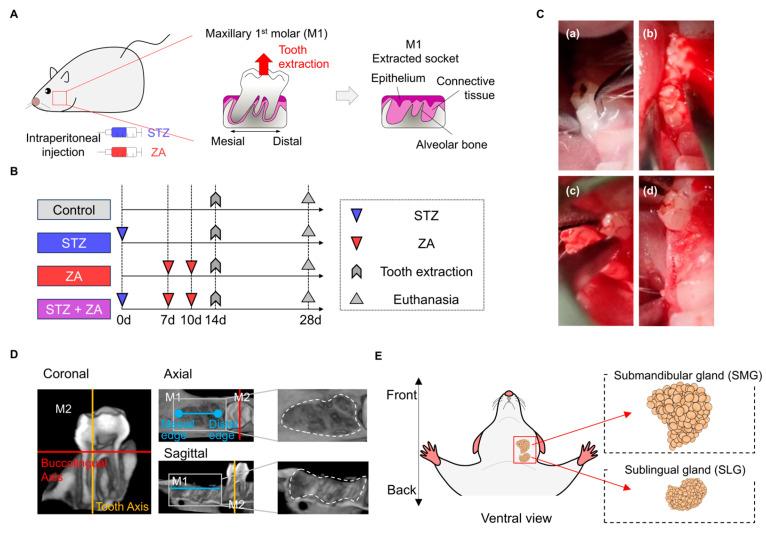
Experimental design and schematic representation of the diabetes-associated bisphosphonate-related osteonecrosis of the jaw (BRONJ) model. (**A**) Schematic illustration of the diabetes-associated BRONJ model, induced by streptozotocin (STZ) and zoledronic acid (ZA). (**B**) Experimental schedule of the Control, STZ, ZA, and STZ + ZA groups. (**C**) Tooth extraction procedure: (**a**) transection of the periodontal ligament; (**b**) tooth dislocation; (**c**) tooth extraction; (**d**) post-extraction socket. (**D**) Definition of the region of interest (ROI) for microfocus X-ray computed tomography (µCT) in the extraction socket: coronal plane showing the tooth axis (yellow line) and buccolingual axis (red line) of the extracted second molar (M2); axial plane showing the mesial and distal edges (blue dots) of the first molar (M1) and buccolingual axis (red line); sagittal plane showing the mesiodistal axis (blue line) of M1 and M2, along M2 tooth axis (yellow line). Right panels in the coronal and sagittal planes show magnified views of the extraction socket. The region surrounded by dotted lines indicates the area of interest for subsequent analysis. (**E**) Schematic ventral view of a rat showing the position of the submandibular gland (SMG) and sublingual gland (SLG). The SMG and SLG were excised en bloc from the cervical region for subsequent morphological and histological analyses.

To induce diabetes, four-week-old rats were intraperitoneally administered streptozotocin (STZ; Nacalai Tesque, Kyoto, Japan) dissolved in a citric acid solution at a dose of 65 mg/kg (Figure 1A), as previously described [26]. To induce BRONJ, zoledronic acid (ZA; Zometa, Novartis, Basel, Switzerland) was administered at 60 µg/kg on Days 7 and 10 (Figure 1A), according to a previously reported protocol [27]. The 16 rats were randomly divided into four groups: Control, STZ, ZA, and STZ + ZA (Figure 1B; *n* = 4 per group), with the experimental unit defined as a single animal. Maxillary first molars (M1) were extracted on Day 14 (Figure 1B,C). Tooth extraction was performed by severing the periodontal ligament with an explorer (Figure 1C(a)), grasping the tooth with forceps (Figure 1C(b)), dislocating and extracting the tooth (Figure 1C(c)), and applying pressure to the extraction socket to achieve hemostasis (Figure 1C(d)). Twenty-eight days after extraction, the animals were euthanized, and perfusion fixation was performed using 4% paraformaldehyde (4% PFA; Muto Pure Chemicals, Tokyo, Japan). The maxillae were harvested en bloc for subsequent analysis.

### 2.3. Evaluation of Diabetic Status

To confirm diabetes induction, blood was collected from the tail vein of all four groups (Control, STZ, ZA, and STZ + ZA) on Days 0, 3, 7, 14, 21, and 28. Blood glucose (GLU) levels were measured over time using Labo Gluco (Research & Innovation Japan Inc., Chiba, Japan). Furthermore, to assess biochemical changes, blood samples were collected on Day 28 and analyzed for glycated albumin (GA), lipid parameters (total cholesterol [T-CHO], triglycerides [TG], low-density lipoprotein cholesterol [LDL-C], high-density lipoprotein cholesterol [HDL-C], non-esterified fatty acids [NEFA], and total ketone bodies [T-KB]), and renal function markers (creatinine [CRE] and urea nitrogen [UN]) according to the protocol of Oriental Yeast Nagahama Life Science Laboratory (Shiga, Japan). Based on previous reports indicating that STZ-induced diabetic rats exhibit suppressed weight gain [28], body weight was measured every three days using a New-Shinobu balance (D7059-6; Ishida Co., Ltd., Osaka, Japan).

### 2.4. Morphological Analysis

The size of the extracted tooth and the epithelialization of the extraction socket were evaluated using a stereo microscope (SZ61, Olympus, Tokyo, Japan). Microfocus X-ray computed tomography (SkyScan μCT, Bruker, Billerica, MA, USA) was performed under the following conditions: 85 kV, 65 µA, high resolution = 1944 × 1536, rotation step = 0.2°, and an average frame count of four images. Plane orientation was defined as follows (Figure 1D): the coronal plane was determined by the maxillary second molar (M2) tooth axis (yellow line) and the buccolingual axis (red line) of M2; the axial plane was determined by a line connecting the mesial and distal edges of the M1 extraction socket (blue line) and the buccolingual axis (red line) of M2; and the sagittal plane was defined by the M2 tooth axis (yellow line) and the mesiodistal axis (blue line) of the M1 extraction socket. Three-dimensional (3D) reconstruction and analysis of bone morphometric parameters, including bone volume/total volume (BV/TV) and trabecular number (Tb.N) within the extraction sockets, were performed using CTvox (ver. 3.3.0; Bruker) and CTAn (ver. 1.17.7.2; Bruker). In addition, 3D reconstruction models of the extraction sockets were generated using 3D Slicer (version 5.6.1; Brigham and Women’s Hospital, Boston, MA, USA).

### 2.5. Histological Analysis

Maxilla samples were fixed in 4% PFA for 24 h. Decalcification was performed at 4 °C for two weeks using an ethylenediaminetetraacetic acid solution (FUJIFILM Wako Pure Chemical, Tokyo, Japan). Following the protocol of KAC Co., Ltd. (Kyoto, Japan), the samples were paraffin-embedded, sectioned at 2.5 µm, and stained with hematoxylin and eosin (H-E). A system microscope (BX41, Olympus) combined with a DP22 digital imaging system and a DP2-SAL standalone controller (Olympus) was used for image acquisition. For each H-E image, the percentage of inflammatory cells was calculated as the mean value of three randomly selected high-magnification fields within the connective tissue of the extraction socket. Empty osteocyte lacunae (EOL), defined as lacunae without any cellular components or nuclei, were quantified using Fiji (ImageJ version 1.54p, National Institutes of Health, Bethesda, MD, USA). We quantified both (1) the overall percentage of EOLs to assess total osteocyte death and (2) the contiguous necrotic bone area to evaluate the size of established necrotic lesions. For the measurement of necrotic bone area, regions containing 10 or more consecutive EOLs were defined as necrotic bone regions, and their areas were calculated using Fiji, following a previously reported method [29].

Samples of the submandibular gland (SMG) and sublingual gland (SLG) were collected (Figure 1E). Each tissue was fixed at 4 °C for 24 h and sequentially treated with 10%, 20%, and 30% sucrose in ultrapure water to prevent ice crystal formation. Tissues were embedded in frozen blocks using Super Cryoembedding Medium (SECTION-LAB Co., Ltd., Yokohama, Japan), and 10 µm frozen sections were prepared using a cryostat (CM3050S; Leica Biosystems, Deer Park, IL, USA) according to the Kawamoto method [30]. Images were captured using a polarizing light microscope (BX41, Olympus). For morphometric evaluation, 108 grid points were overlaid on each image, and the number of points corresponding to serous acini of the SMG and mucous acini of the SLG was counted. The volume fraction (Vv) was calculated using the formula: Vv = number of points hitting the structure/total number of test points (*n* = 108), as previously reported [31]. The average Vv value was obtained from three randomly selected fields within each section.

### 2.6. Saliva Test

To induce salivation, rats were fasted starting at 8:00 p.m. on the day prior to saliva collection, and saliva was collected beginning at 9:00 a.m. the following morning. Pilocarpine hydrochloride (28008-31; Nacalai Tesque) was diluted with ultrapure water to a concentration of 0.1 mg/mL and intraperitoneally administered at a dose of 0.86 mg/kg. Saliva was collected using micropipettes continuously for one hour. The collected saliva volume was categorized as High (>1.0 mL), Medium (0.5–1.0 mL), or Low (<0.5 mL). To assess biochemical changes, saliva samples were collected from each group on Day 28. Blood glucose markers (GLU, GA) and lipids (T-CHO, TG, LDL-C, HDL-C, T-KB, NEFA) and renal function markers (UN, CRE) were analyzed according to the protocol of Oriental Yeast Nagahama Life Science Laboratory. During the first screening, detectability was assessed and categorized into three groups: Category 1 (mostly detectable), Category 2 (partially detectable), and Category 3 (undetectable). In the second screening, the concentrations of detectable markers were compared among the groups. The molecular weights and molecular formulas of the analyzed biochemical markers were summarized from previously published literature.

### 2.7. Statistical Analysis

All statistical analyses were performed using GraphPad Prism ver. 10.4.1 (GraphPad Software, Boston, MA, USA). Data are expressed as mean ± standard deviation (SD). Comparisons between two groups were conducted using an unpaired Student’s *t*-test. Comparisons among three or more groups were performed using one-way analysis of variance (ANOVA); Tukey’s multiple comparison test was applied for homogeneous variances, whereas the Brown–Forsythe test was used for heterogeneous variances. The Kruskal–Wallis test served as a nonparametric alternative for non-normally distributed data. A *p*-value < 0.05 was considered statistically significant (* *p* < 0.05; ** *p* < 0.01; *** *p* < 0.001; **** *p* < 0.0001).

## 3. Results

### 3.1. Establishment of a Diabetes Model

**Figure 2:**
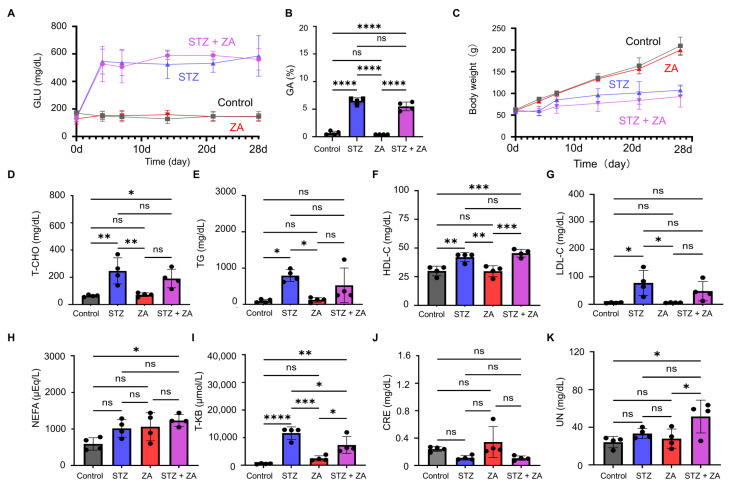
Establishment of the diabetes model. (**A**) Sequential quantitative analysis of blood glucose (GLU) levels. (**B**) Quantitative analysis of blood glycated albumin (GA). (**C**) Sequential quantitative analysis of body weight. (**D**–**K**) Quantitative analyses of metabolic parameters, including total cholesterol (T-CHO), triglycerides (TG), high-density lipoprotein cholesterol (HDL-C), low-density lipoprotein cholesterol (LDL-C), non-esterified fatty acids (NEFA), total ketone bodies (T-KB), creatinine (CRE), and urea nitrogen (UN). Data are presented as the mean ± SD (*n* = 4 per group). * *p* < 0.05, ** *p* < 0.01, *** *p* < 0.001, **** *p* < 0.0001; ns, not significant.

Sequential blood tests revealed an increase in blood glucose levels on day 4 after STZ administration (Figure 2A). Furthermore, an increase in GA levels was observed on day 28 (Figure 2B). Consistent with previously reported diabetic models [28], body weight gain was suppressed in the STZ group (Figure 2C). The STZ + ZA group exhibited abnormalities similar to those observed in the STZ group, whereas the ZA group did not induce abnormalities and showed a profile comparable to the Control group (Figure 2A–C). Significant abnormalities were also observed in lipid-related markers in the STZ and STZ + ZA groups, including T-CHO, TG, HDL-C, LDL-C, NEFA, and T-KB (Figure 2D–I). Among the renal function markers, CRE remained within the normal range across all groups, whereas blood urea UN was elevated in the STZ and STZ + ZA groups (Figure 2J,K). The ZA group showed almost no abnormalities across these lipid and renal parameters (Figure 2J,K).

### 3.2. Changes in Soft Tissue of Extraction Sockets Following ZA Administration in a Diabetes Model

**Figure 3:**
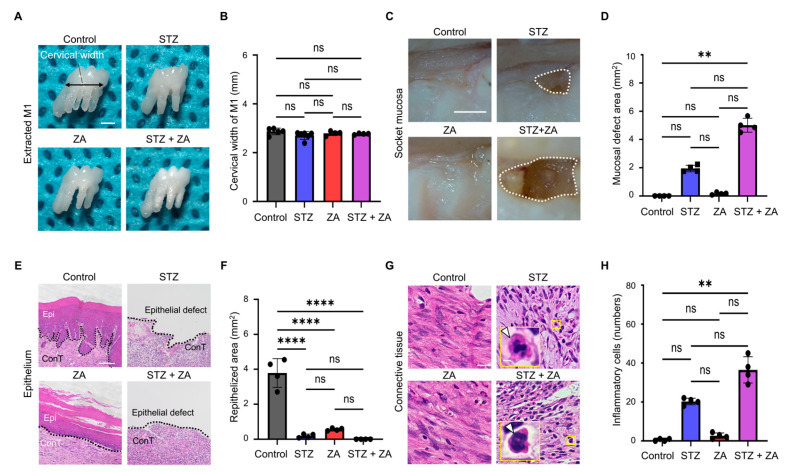
Macroscopic and histological evaluation of mucosal healing after tooth extraction. (**A**) Stereomicroscopic image of the extracted first molar (M1). Double-headed arrow: cervical width of M1. Scale bar, 1 mm. (**B**) Quantitative analysis of cervical width of M1. (**C**) Representative stereomicroscopic images of the extraction socket at 28 d. Dotted line: unhealed mucosal defect area. Scale bar, 1 mm. (**D**) Quantitative analysis of the mucosal defect area at 28 d. (**E**) High-magnification Hematoxylin and eosin (H-E) images of the epithelium (Epi). Scale bar, 100 μm. ConT: connective tissue. (**F**) Quantitative analysis of re-epithelized area. (**G**) High-magnification H-E images of the connective tissue. White arrowheads: inflammatory cells. Scale bars: 20 μm (overview) and 12 μm (magnified view of the boxed area). (**H**) Quantitative analysis of inflammatory cells. Data are presented as the mean ± SD (*n* = 4 per group). ** *p* < 0.01, **** *p* < 0.0001; ns, not significant.

Comparison of the cervical width of the extracted M1 revealed no significant differences among groups (Figure 3A,B), indicating comparable initial wound sizes and ensuring a standardized model for healing assessment. Stereomicroscopic examination revealed enlargement of the epithelial defect in the extraction socket (Figure 3C,D). Histological analysis showed a reduction in the area of epithelial re-epithelialization (Figure 3E,F). In the connective tissue, an increase in inflammatory cells (arrowhead) was observed (Figure 3G,H).

### 3.3. Changes in Hard Tissue of the Extraction Socket Following ZA Administration in a Diabetes Model

**Figure 4:**
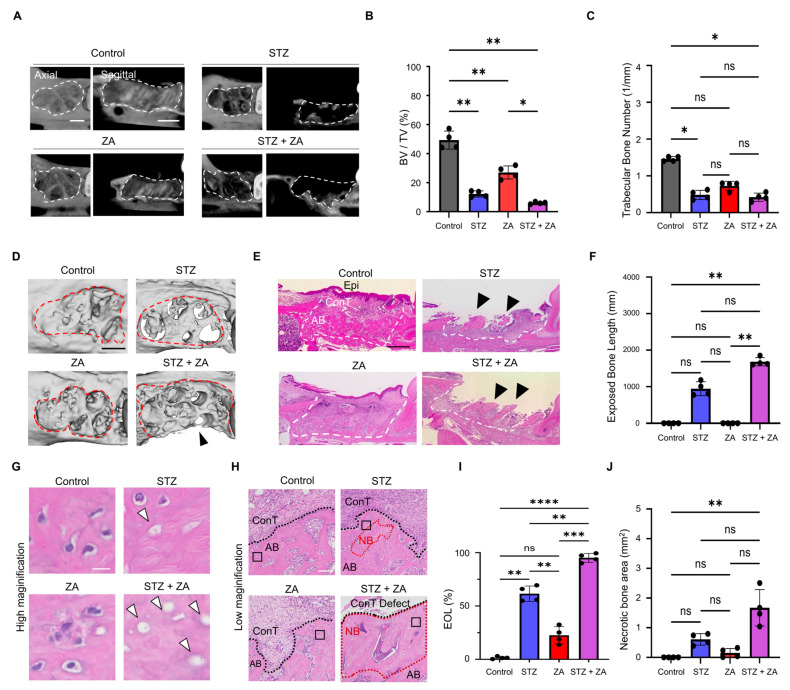
µCT and histological evaluation of alveolar bone healing. (**A**) Representative µCT images of extraction sockets at 28 d. Dashed lines: extraction socket. Scale bar, 1 mm. (**B**) Quantitative analysis of bone volume/tissue volume (BV/TV) in the extraction socket based on µCT data. (**C**) Quantitative analysis of trabecular bone number in the extraction socket based on µCT data. (**D**) Three-dimensional reconstructed µCT images showing bone morphology and defects (dashed line: extraction socket, arrowhead: cortical bone defect). Scale bar, 1 mm. (**E**) Representative low-magnification H-E images of the entire extraction socket. Dashed lines: extraction socket. Black arrowheads: bone exposure. Scale bar, 1 mm. (**F**) Quantitative analysis of exposed bone length. (**G**) Representative high-magnification H-E images of the bone. White arrowheads: empty osteocyte lacunae (EOL) frequently observed in the STZ + ZA group, suggesting bone necrosis. Scale bar, 20 μm. (**H**) Representative H-E-stained coronal sections of extraction sockets at 28 d. The black squares indicate the regions shown at high magnification in (**G**). The dashed line indicates the border between the alveolar bone (AB) and the connective tissue (ConT). Necrotic bone (NB) is observed in the STZ and STZ + ZA groups. Scale bar, 100 μm. (**I**) Quantitative analysis of EOL (%) as EOL/total osteocyte lacunae. (**J**) Quantitative analysis of necrotic bone area (regions containing 10 or more consecutive EOLs). Data are presented as the mean ± SD (*n* = 4 per group). * *p* < 0.05, ** *p* < 0.01, *** *p* < 0.001, **** *p* < 0.0001; ns, not significant.

µCT analysis revealed increased radiolucency of the extraction sockets in the STZ, ZA, and STZ + ZA groups compared with the Control group (Figure 4A). Correspondingly, both BV/TV and Tb. N values were markedly decreased (Figure 4B,C). 3D reconstruction demonstrated expanded bone destruction surrounding the extraction sockets, with buccal cortical bone defects (black arrowhead) observed in the STZ + ZA group (Figure 4D). Furthermore, bone exposure was observed in the extraction socket in the STZ group, with a greater extent of exposure in the STZ + ZA group (Figure 4E,F). To evaluate bone pathology, we compared across groups (1) the percentage of EOLs among total osteocyte lacunae and (2) the necrotic bone area, defined as regions containing 10 or more consecutive EOLs. Numerous EOLs were observed within the alveolar bone (Figure 4G,H). Quantitative analysis showed that the proportion of EOLs among total lacunae increased in the STZ group compared with the Control group and further increased in the STZ + ZA group (Figure 4I). Similarly, the extent of necrotic bone areas—defined as a contiguous series of 10 empty osteocyte lacunae—was increased in the STZ + ZA group (Figure 4J).

### 3.4. Changes in SGs Following ZA Administration in a Diabetes Model

**Figure 5:**
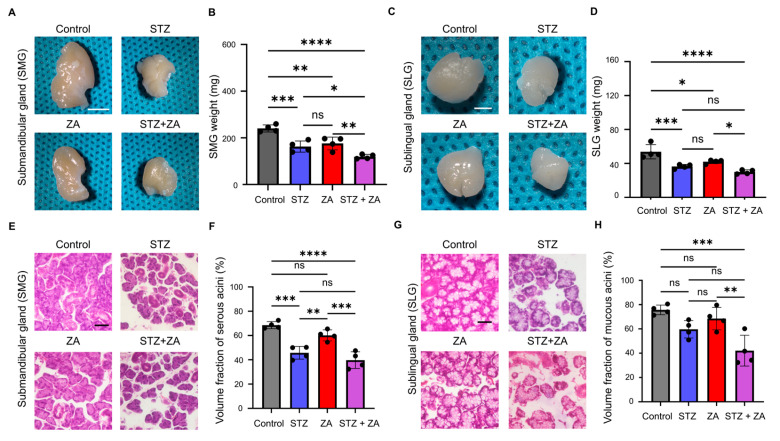
Morphological and weight changes of the submandibular (SMG) and sublingual glands (SLG) in each group. (**A**) Representative stereomicroscopic images of the SMG from each group. Scale bar, 500 μm. (**B**) Quantitative analysis of SMG weight. (**C**) Representative stereomicroscopic images of the SLG. Scale bar, 200 μm. (**D**) Quantitative analysis of the SLG weight. (**E**) Representative H-E-stained sections of SMG from each group showing serous acini morphology. Scale bar, 100 μm. (**F**) Quantitative analysis of the volume fraction (Vv) of serous acini in SMG. (**G**) Representative H-E-stained sections of SLG showing mucous acini morphology. Scale bar, 100 μm. (**H**) Quantitative analysis of the Vv of mucous acini in SLG. Data are presented as the mean ± SD (*n* = 4 per group). * *p* < 0.05, ** *p* < 0.01, *** *p* < 0.001, **** *p* < 0.0001; ns, not significant.

Stereomicroscopic examination demonstrated significant reductions in both size and weight of the SMG and SLG (Figure 5A–D). Among the experimental groups, SMG and SLG size and weight were reduced in the STZ, ZA, and STZ + ZA groups compared with the control group, with the STZ + ZA group showing the most marked decrease. Histological examination revealed a decrease in the Vv of serous acini in the SMG (Figure 5E,F) and mucous acini in the SLG (Figure 5G,H).

### 3.5. Changes in Saliva Following ZA Administration in a Diabetes Model

**Figure 6:**
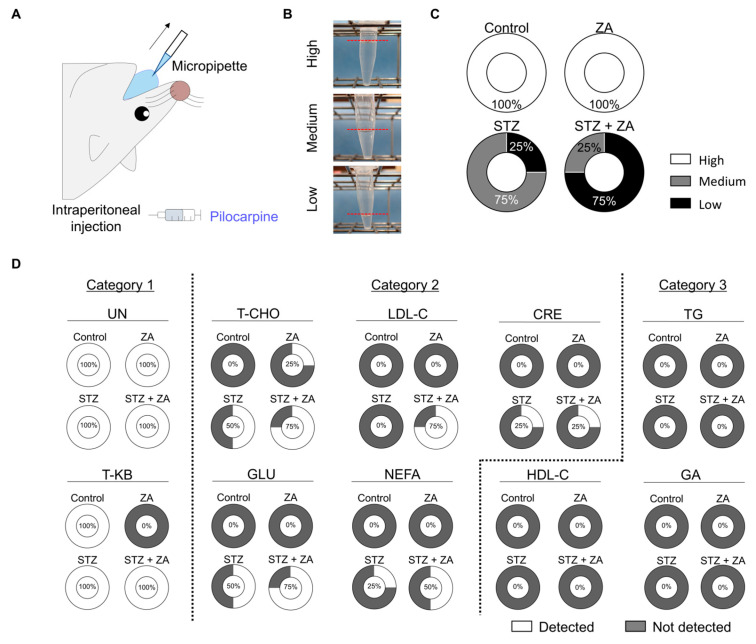
First screening of diabetes-related salivary biochemical markers. (**A**) Schematic illustration of the pilocarpine-induced salivation procedure. Salivary secretion was stimulated via intraperitoneal injection of pilocarpine. Subsequently, secreted saliva was collected using a micropipette. (**B**) Representative images of salivation levels classified into three categories—High, Medium, and Low—based on the amount of collected saliva. The red dashed lines indicate the height of the liquid meniscus. (**C**) Categorical distribution of salivation levels among groups. (**D**) Detection frequencies of 10 salivary biochemical markers across all groups: UN, T-KB, T-CHO, GLU, LDL-C, NEFA, CRE, HDL-C, TG, GA. Each circle shows the percentage of animals in which the marker was above detection limits. Gray-filled circles indicate “Not detected”; white circles represent “Detected” (*n* = 4 per group).

Pilocarpine was administered to rats in each group to induce salivation, and saliva was collected (Figure 6A). The collected saliva volume was classified as High (>1.0 mL), Medium (0.5–1.0 mL), or Low (<0.5 mL) (Figure 6B). All samples were classified as High in the Control and ZA groups, whereas Medium samples predominated in the STZ group and Low samples predominated in the STZ + ZA group (Figure 6C), indicating reduced salivary secretion.

Screening of salivary biochemical markers in each group revealed that UN and T-KB were detectable in most samples (Figure 6D; Category 1). For T-CHO, GLU, LDL-C, NEFA, and CRE, detection was achieved in approximately 25–75% of samples in the STZ + ZA group (Category 2). In contrast, TG, HDL-C, and GA were below the detection limit (not detected [ND]) in all samples (Category 3).

### 3.6. Second Screening of Saliva Tests

Quantitative analysis was performed for salivary markers detected during the first screening, in which the markers were classified into Groups 1 and 2 (Table 1). Increased mean values of UN and T-KB were observed in the STZ + ZA group. Conversely, for T-CHO, GLU, LDL-C, NEFA, and CRE, increases were observed in some samples; however, the differences were unclear because of the presence of undetectable (ND) samples (Table 1). Furthermore, analysis of the molecular weights of the examined markers, summarized from previous reports, revealed that UN and T-KB had lower molecular weights than the other markers (Table 2).

## 4. Discussion

In this study, we successfully established a diabetic BRONJ rat model by combining STZ-induced diabetes with zoledronic acid administration, followed by tooth extraction. Using this model, we aimed to obtain fundamental insights into the development of salivary diagnostic approaches by examining salivary gland alterations and conducting an exploratory screening of salivary biochemical markers.

To establish a diabetic BRONJ model, STZ-induced diabetes was combined with ZA administration. The STZ dose used in this study (65 mg/kg) effectively induced a severe diabetic state, as previously reported [32,33]. STZ administration resulted in elevated blood glucose (Figure 2A) and increased GA levels (Figure 2B), along with suppressed weight gain (Figure 2C), consistent with previous findings in STZ-induced diabetic models [28,34]. Furthermore, blood tests in diabetes frequently reveal dyslipidemia [35] and renal dysfunction [36]. In this study, elevated levels of lipid-related markers (T-CHO, TG, HDL-C, LDL-C, NEFA, and T-KB) (Figure 2D–I) and renal function markers (CRE and UN) (Figure 2J,K) were observed, indicating that STZ-induced diabetic rats exhibited both dyslipidemia and renal dysfunction. According to the AAOMS Position Paper, the diagnostic criteria for BRONJ include a history of antiresorptive agent use, jawbone exposure, and the absence of radiation therapy or bone metastasis, suggesting that necrotic bone is observed in severe cases [6]. In the extraction sockets of the STZ + ZA group, enlargement of epithelial defects (Figure 3C–F), inflammatory cell infiltration in connective tissue (Figure 3G,H), bone exposure within the extraction socket (Figure 4E,F), and increased necrotic bone with EOLs (Figure 4G–J) were observed. These results demonstrate that our model successfully reproduced the key pathological features of severe, diabetes-associated BRONJ.

To assess the effects of diabetes, ZA, and BRONJ on the salivary glands, macroscopic appearance and weight were examined in both the SMG and SLG. Diabetes caused a reduction in SMG size (Figure 5A) and decreased SMG weight (Figure 5B). Similarly, the SLG showed reduced size (Figure 5C) and decreased weight (Figure 5D). Histologically, the rat SMG is predominantly serous [37], whereas the SLG is mainly mucous [38]. Notably, the Vv decreased in both the serous acini of the SMG (Figure 5E,F) and the mucous acini of the SLG (Figure 5G,H). Diabetes has been reported to cause reduced SG weight, acinar cell atrophy, and enlarged intercellular spaces [39]. Although reports on the direct effects of ZA on SGs are limited, salivary secretion tends to decrease in ZA-treated patients or BRONJ patients [40]. In this study, STZ administration also led to reduced salivary secretion, which was further aggravated by combined STZ + ZA treatment (Figure 6C). These findings indicate that our model reproduced the atrophic changes in SG tissue and the decreased salivary secretion observed in diabetes, with ZA potentially exacerbating these effects.

Although salivary secretion decreased, saliva samples were successfully collected from all rats (Figure 6C). Therefore, salivary testing was performed to screen secreted biochemical markers. Among the 10 examined markers, UN and T-KB were detectable in most samples, whereas T-CHO, GLU, LDL-C, NEFA and CRE were detectable in approximately half of the samples (Figure 6D). UN (60 Da) and T-KB (104 Da) are low-molecular-weight compounds (Table 2) and may transfer relatively easily into saliva. Conversely, HDL-C, TG and GA were below the detection limit in all samples in the STZ + ZA group (Figure 6D), suggesting that these markers may have large molecular weights, making their transfer into saliva more difficult (Table 2). Molecules present in saliva migrate through active transport, passive diffusion, and tissue permeability within the oral cavity. Large molecules, such as high-molecular-weight proteins, have difficulty passing through the SG stroma and epithelium, whereas small-molecule metabolites and hormones are transferred more easily [41]. Based on this, low-molecular-weight compounds like UN and T-KB were proposed as primary candidates for salivary testing, whereas T-CHO, GLU, LDL-C, NEFA and CRE were suggested as secondary candidates for first screening. Subsequent analysis of marker expression among the groups revealed elevated levels of UN and T-KB in the diabetes + ZA group (Table 1). Although few studies have established these as salivary biomarkers, several reports provide supporting evidence. UN has been proposed as a potential salivary biomarker for renal function [42], although its detection is often difficult unless concentrations exceed a certain threshold [43]. Reports on T-KB as a salivary biomarker are limited; however, their components β-hydroxybutyrate and acetone have been suggested as useful indicators for evaluating ketosis in salivary testing [44]. Overall, these findings suggest the potential utility of UN and T-KB as salivary markers for diabetes and diabetes-associated BRONJ.

This study has several limitations. First, the evaluation was performed at a single time point (day 28), precluding detailed analysis of the temporal progression of diabetes and BRONJ. Second, the detectable markers in saliva were limited to diabetes-related biochemical markers; other potentially relevant molecules, such as mRNAs related to inflammation or oxidative stress, may also be involved. Finally, as this was a study conducted in a rat model, the translation of these findings to the complex clinical pathology in humans requires further investigation. Nevertheless, this study screened salivary biomarkers using a diabetic rat BRONJ model and demonstrated that UN and T-KB levels were elevated in diabetic BRONJ.

## 5. Conclusions

In this study, we established a rat model of BRONJ complicated by diabetes through the combination of STZ-induced diabetes with zoledronic acid administration. This model reproduced the key pathological features of severe BRONJ, including epithelial defects, inflammatory cell infiltration, and necrotic bone formation. Diabetes and ZA administration induced atrophic changes in the SMGs and SLGs, resulting in reduced salivary secretion. Biochemical screening of saliva revealed significantly elevated levels of low-molecular-weight markers such as UN and T-KB in the diabetic and diabetic + ZA groups. These findings suggest that UN and T-KB may serve as potential salivary biomarkers reflecting metabolic and renal dysfunction in diabetic BRONJ. Overall, this study provides foundational insights into the identification of salivary biomarkers for diabetic BRONJ and may contribute to the development of noninvasive salivary monitoring methods.

## Figures and Tables

**Table 1:** Second screening: Summary of salivary biochemical parameters in each experimental group.

	Control		STZ		ZA		STZ + ZA	
	MEAN	Range	MEAN	Range	MEAN	Range	MEAN	Range
UN (mg/dL)	11.4	9.9–12.3	19.6	10.6–20.4	9.75	9.1–10.8	27.6	11.3–40.3
T-KB (μmol/L)	8.25	3.0–15.0	294	8.0–482.0	ND	ND	419	19.0–640.0
T-CHO (mg/dL)	ND	ND	3	ND–4.0	0.5	ND–2.0	3.7	ND–6.0
GLU (mg/dL)	ND	ND	0.5	ND–2.0	ND	ND	2	ND–3.0
LDL-C (mg/dL)	ND	ND	ND	ND	ND	ND	1.7	ND–3.0
NEFA (μEq/L)	ND	ND	ND	ND	ND	ND	26.4	ND–105.5
CRE (mg/dL)	ND	ND	ND	ND	ND	ND	0.06	ND–0.06

“ND” indicates a value below the detection limit of the assay.

**Table 2:** Representative molecular weights of biochemical markers.

Marker	Molecular Weight (Da)	Molecular Formula
UN (mg/dL)	60	CH_4_N_2_O
T-KB (μmol/L)	104	C_4_H_8_O_3_/C_4_H_6_O_3_
T-CHO (mg/dL)	387	C_27_H_46_O
GLU (mg/dL)	180	C_6_H_12_O_6_
LDL-C (mg/dL)	~2.3 × 10^6^	–
NEFA (μEq/L)	256	C_16_H_32_O_2_
CRE (mg/dL)	113	C_4_H_7_N_3_O
HDL-C (mg/dL)	~3.5 × 10^5^	–
TG (mg/dL)	885	C_57_H_104_O_6_
GA (%)	66,500	–

## Data Availability

The original contributions presented in this study are included in the article. Further inquiries can be directed to the corresponding author.

## Abbreviations

The following abbreviations are used in this manuscript:

ANOVA	Analysis of variance
BRONJ	Bisphosphonate-related osteonecrosis of the jaw
BV/TV	Bone volume to total volume ratio
CRE	Creatinine
EOL	Empty osteocyte lacunae
GA	Glycated albumin
GLU	Glucose
HDL-C	High-density lipoprotein cholesterol
H-E	Hematoxylin and eosin
LDL-C	Low-density lipoprotein cholesterol
M1	Maxillary first molar
M2	Maxillary second molar
ND	Not detected
NEFA	Non-esterified fatty acids
PFA	Paraformaldehyde
SD	Standard deviation
SGs	Salivary glands
SLG	Sublingual gland
SMG	Submandibular gland
STZ	Streptozotocin
Tb.N	Trabecular number
TG	Triglyceride
T-CHO	Total cholesterol
T-KB	Total ketone bodies
UN	Urea nitrogen
Vv	Volume fraction
ZA	Zoledronic acid
